# Low Dose Infliximab for Prevention of Postoperative Recurrence of Crohn’s Disease: Long Term Follow-Up and Impact of Infliximab Trough Levels and Antibodies to Infliximab

**DOI:** 10.1371/journal.pone.0144900

**Published:** 2015-12-15

**Authors:** Dario Sorrentino, Marco Marino, Themistocles Dassopoulos, Dimitra Zarifi, Tiziana Del Bianco

**Affiliations:** 1 IBD Center, Division of Gastroenterology, Virginia Tech-Carilion School of Medicine, Roanoke, Virginia, United States of America; 2 Department of Clinical and Experimental Medical Sciences, University of Udine School of Medicine, Udine, Italy; 3 Baylor Center for IBD, Baylor University Medical Center, Dallas, Texas, United States of America; CWRU/UH Digestive Health Institute, UNITED STATES

## Abstract

**Objective:**

In patients with postoperative recurrence of Crohn’s disease endoscopic and clinical remission can be maintained for up to 1 year with low infliximab doses (3 mg/Kg). However, in theory low-dose infliximab treated patients could develop subtherapeutic trough levels, infiximab antibodies, and might loose response to therapy. To verify this hypothesis infliximab pharmacokinetics and clinical/endoscopic response were checked in a group of patients treated in the long term with low infliximab doses.

**Design:**

Infliximab antibodies, infliximab levels, highly-sensitive CRP and fecal calprotectin were measured during the 8-week interval in 5 consecutive patients in clinical (Crohn’s Disease Activity Index < 150) and endoscopic (Rutgeerts scores 0–1) remission after one year of therapy with infliximab 3 mg/Kg. For comparison with reported standards, infliximab pharmacokinetics and inflammatory parameters were also tested in 6 Crohn’s disease patients who did not undergo surgery and who were in clinical remission while on infliximab 5 mg/Kg. Patients on low infliximab dose also underwent colonoscopy after 18 additional months of therapy.

**Results:**

Highly sensitive CRP and fecal calprotectin increased in all patients during the 8-week interval. Infliximab trough levels were lower in patients treated with the low dose compared to controls (mean±SE: 2.0±0.3 vs 4.75±0.83 μg/mL respectively p<0.05). Infliximab antibodies were present in two of the subjects treated with low infliximab dose and in none of the controls. However, in low dose-treated patients after 18 additional months of therapy endoscopy continued to show mucosal remission and none of them developed clinical recurrence or side effects.

**Conclusions:**

Patients treated with low infliximab doses had lower trough levels compared to patients treated with 5 mg/Kg and some developed antibodies to infliximab. However, low infliximab doses sustained clinical and endoscopic remission for a total of 30 months of treatment.

## Introduction

Since 2006, the monoclonal anti-TNF-α antibodies infliximab and adalimumab have been shown in several studies to be highly effective in preventing post-operative recurrence [POR] of Crohn’s disease [CD] [1]. Initial studies from our group showed that maintenance infliximab is effective in preventing POR in the long term [2]–a finding recently confirmed by others [3]. However—as in patients who have not undergone surgery—the long-term management of CD patients with biologics after surgery incurs significant costs and safety risks [4–9]. Stopping infliximab has been proposed by some authors [10,11] however this is followed by prompt endoscopic disease relapse [2], eventually leading to clinical recurrence [3]. To address this issue, we proposed in a pilot study a dose titration strategy, with the goal of finding the minimal effective dose of infliximab in patients with endoscopic recurrence after surgery [2]. We showed that a dose of 3 mg/Kg was capable of inducing and maintaining endoscopic and clinical remission for up to 1 year in all patients [2].

A theoretical issue in adopting a low dose strategy in the long term is the formation of antibodies to infliximab [ATI]—as a result of low infliximab trough levels [ITL]—an event that could also lead to loss of response and/or infusion reactions [12–14]. The generally accepted therapeutic threshold for ITL has been reported to be ≥3 μg/mL [12, 15, 16].

The goal of the present study was to clarify this issue and provide extended follow-up data on patients maintained on low-dose infliximab to prevent POR. For this purpose we measured ITL, ATI as well as markers of disease activity in 5 consecutively selected patients with proven POR maintained in clinical and endoscopic remission with 3 mg/kg doses of infliximab for one year. To compare results with those reported in the literature for standard infliximab doses [12,15], ITL, ATI and inflammation markers were also measured in 6 controls (CD patients who did not undergo surgery and in clinical remission treated with infliximab 5 mg/Kg).

## Methods

### Study design

**Fig 1:**
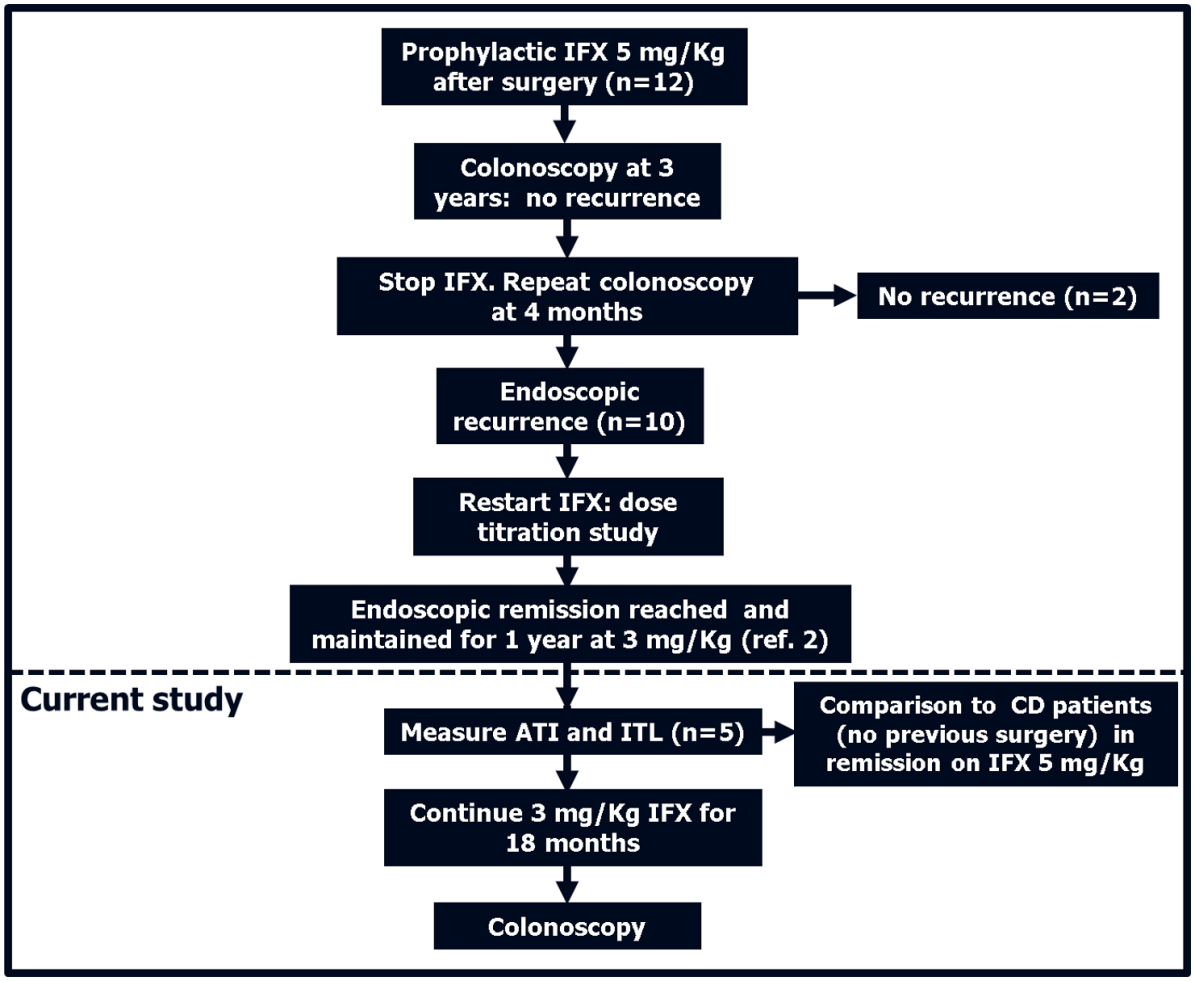
Study Design. Patients subjected to prophylactic infliximab [IFX] 5 mg/Kg after surgery and in full remission after 3 years of therapy showed endoscopic recurrence in 83% of cases when the medication was stopped. Those with recurrence (n = 10) re-started IFX in a dose optimization study in which we showed that 3 mg/Kg were sufficient to re-induce and maintain endoscopic remission for 1 year in all patients (ref.2). Five of these ten patients participated in the current study. Antibodies To Infliximab [ATI] and Infliximab Trough Levels [ITL] were measured and compared to those of CD patients who did not undergo surgery in remission on a standard 5 mg/Kg IFX dose. After an additional 18 months the patients treated with 3 mg/Kg IFX underwent colonoscopy.

Five of the ten patients subjected to the dose titration study [2] were consecutively enrolled to participate in the current study (Fig 1). They all presented endoscopic relapse when the standard dose (5 mg/Kg) infliximab—initiated immediately after surgery and continued for 3 years—was stopped for 4 months [2]. Mucosal healing was then re-induced with 3 mg/Kg infliximab [2]. When enrolled in the current study they were all in clinical (Crohn’s Disease Activity Index [CDAI] < 150) [17] and endoscopic remission (Rutgeerts score 0–1) [18] after one year of infliximab treatment at 3 mg/Kg. Individual Rutgeerts scores at enrollment were 0,1,1,1,0 in the patients progressively numbered 1–5 (Table 1). Solely for the purpose to compare ITL, ATI and inflammatory markers in our study with those reported in the literature using standard infliximab doses [12,15] six controls (CD patients in clinical remission [CDAI < 150] who did not undergo surgery) treated with 5 mg/Kg were also included in the study. None of the study subjects and the controls were concomitantly treated with immunomodulators. All patients were asked to provide a stool sample at weeks 4 and 8 of the 8-week infliximab therapeutic interval for the measurement of fecal calprotectin [FC], performed by a commercially available ELISA test (Calprest, Eurospital, Trieste-Italy) after protein extraction on a weighted stool sample. Blood was taken immediately before, immediately after the infusion and at 4 weeks. In these samples, infliximab concentrations and ATI were measured by homogenous mobility shift assay [19], with limits of detection of 0.91 μg/mL and 3.13 U/mL respectively—while highly sensitive CRP [HS-CRP] was measured by proprietary methods (Prometheus Laboratories Inc, San Diego, CA). These tests were performed during at least three consecutive 8-week therapeutic intervals within the same time frame for all the patients. All the five study subjects on low infliximab doses (but none of the controls) were followed clinically every 6 months and were subjected to colonoscopy after 18 additional months of therapy. The other five patients on low infliximab dose from the original titration study (2) were only followed clinically for an additional 12 months.

**Table 1:** Patient features at enrollment.

Patients	Age/Sex	Disease duration (years)	Disease location	Previous surgery	Smoking status	Infliximab dose (mg/Kg)	Treatment duration
***Study subjects***							
#1	51/M	8	Neo-TI	Yes	Yes	3	12 months
#2	40/F	11	Neo-TI	Yes	No	3	12 months
#3	44/M	12	Neo-Ti	Yes	No	3	12 months
#4	36/M	3	Neo-TI	Yes	No	3	12 months
#5	32/M	4	Neo-TI	Yes	No	3	12 months
***Controls***							
#1C	29/M	6	TI + colon	No	Yes	5	16 months
#2C	43/M	5	TI	No	No	5	8 months
#3C	24/M	7	Left colon	fistula	No	5	12 months
#4C	37/M	4	ileocecal	No	No	5	7 months
#5C	48/M	10	ileocecal	No	No	5	24 months
#6C	32/M	5	TI	No	No	5	14 months

### Ethical considerations

The study was approved by the Ethical Committee of the University of Udine, Italy. Individuals signed a proper informed consent before enrolment.

### Statistical methods

The Student’s *t* test was used to compare infliximab concentrations, HS-CRP and FC levels in study subjects and controls with the significance level set at 5%.

## Results

### Patient features

Table 1 illustrates the main features of the study subjects and controls. The 2 groups did not differ in terms of sex distribution, age (mean±SE: 40.6±3.3 vs 35.5±5.1 years for study subjects and controls respectively) and disease duration (7.6±1.8 vs 6.2±0.8 years). One patient per group was actively smoking at time of enrolment.

### Pharmacokinetics and markers of inflammation

**Fig 2:**
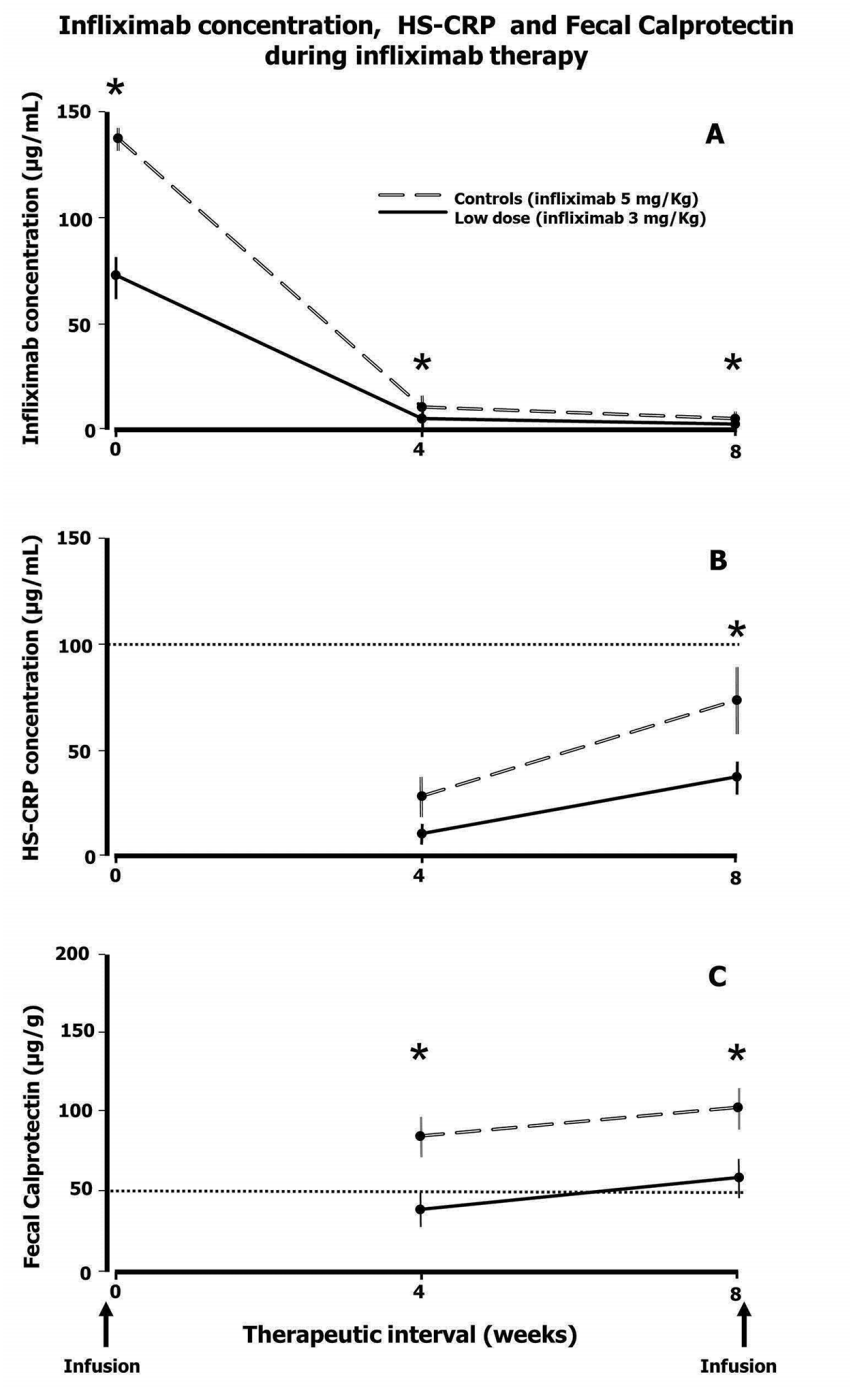
Infliximab concentration (A), Highly Sensitive [HS]–CRP (B) and Fecal Calprotectin (C) during infliximab therapy. Empty long dash lines: control patients (treated with infliximab 5 mg/Kg); Filled lines: study subjects (treated with infliximab 3 mg/Kg). Square dot lines indicate the upper limit of the normal range for HS-CRP and Fecal Calprotectin. Values are reported as mean and standard error (vertical bars). The asterisk denotes a significant difference (p < 0.05) between controls and study subjects at a given time point during the 8-week therapeutic interval.

After the infusion, as expected, infliximab concentrations followed first order kinetics in both controls and study subjects with concentrations at any point in time being approximately half in patients treated with 3 mg/Kg compared to controls (Fig 2A).

In all patients, falling infliximab levels were accompanied by increases in HS-CRP and FC towards the end of the 8 week interval (Fig 2B and 2C).

At any point in time HS-CRP concentrations were higher in controls than in patients treated with 3 mg/Kg (Fig 2B) the difference reaching statistical signaficance for the pre-infusion values. Likewise, mean FC levels in controls were significantly higher than in study subjects and above the upper limit of the normal range at midinterval and before the infusion (Fig 2C).

### ITL and ATI

Mean values ± SE of ITL were 4.75±0.83 μg/mL in controls as opposed to 2.0±0.3 μg/mL in patients treated with infliximab 3 mg/Kg (p<0.05). In 4/5 patients treated with low dose infliximab ITL were lower than 3 μg/mL (Table 2) whereas in all controls ITL were >3 μg/mL. ATI were present in 2/5 patients treated with 3 mg/Kg and in none of the controls. In both ATI-positive patients concentrations were below 10 U/mL (Table 2).

**Table 2:** ITL and ATI in patients treated with infliximab 3 mg/Kg and controls.

	Patient#1	Patient#2	Patient#3	Patient#4	Patient#5	Controls
**ITL (μg/mL)**	4.4±0.8	0.91±0.1	0.91±0.1	1.3±0.3	2.8±0.3	4.75 ±0.83
**ATI (U/mL)**	Negative	5.4±0.3	Negative	7.7±0.8	Negative	Negative

Individual patients ITL and ATI concentrations are the mean ± standard error of 3 consecutive 8-week therapeutic interval sample values measured immediately before infusion. For the 6 controls, all data are pooled together and presented as mean± standard error. Individual samples were tested at least in duplicate. **ITL:** Infliximab Trough Levels. **ATI:** Antibodies To Infliximab

### Clinical follow up and endoscopy

**Fig 3:**
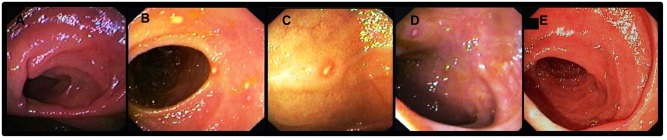
Endoscopy (neo-terminal ileum) after a total of 30 months of therapy with infliximab 3 mg/Kg. Rutgeerts scores were identical to baseline (baseline: one year of treatment with infliximab 3 mg/Kg): 0,1,1,1,0 in patients #1,#2,#3,#4,#5 listed in Table 1, respectively (A-E).

Follow up and endoscopy were undertaken in patients treated with low dose infliximab. After 18.6 ± 5.5 additional months of treatment with infliximab 3 mg/Kg all 5 patients were still in full clinical remission (CDAI < 150). Colonoscopy revealed Rutgeers scores identical to baseline (baseline: one year of treatment with low dose infliximab) and all within endoscopic remission (0,1,1,1,0) (Fig 3A–3E). No infusion reactions or infliximab-related side effects were reported within the entire 30 month treatment period.

The additional 5 patients with POR treated for one year with infliximab 3 mg/Kg originally included in the dose titration study (2) and not enrolled in the current study maintained clinical remission (CDAI < 150) after an additional 12 months of clinical follow up.

## Discussion

Since 2006 a number of studies have shown that anti-TNF agents are effective in preventing POR in CD (reviewed in ref.1). However, long term management of these patients remains an important issue due to costs and potential side effects of these medications [5–9]. Stopping infliximab [10,11] might cause prompt endoscopic relapse [2] which in time might lead to clinical recurrence [3]. Thus, this approach remains the subject of debate [5–9]. Since patients after surgery might bear a low disease burden, we tested lower than standard doses of infliximab in patients with proven endoscopic recurrence—previously maintained in remission with infliximab 5 mg/Kg. We have shown that a dose of 3 mg/kg every 8 weeks appears sufficient to restore and maintain mucosal remission for 1 year in all the studied subjects [2].

However a low dose might lead to low ITL and formation of ATI—an event that could potentially cause loss of response and/or infusion reactions [12–13]. However, it is unclear whether the presence of ATI might affect the clinical outcome in all patients [14, 20, 21]. The threshold ITL below which symptom relapse and inflammation might become more likely has been reported to be 3 μg/mL [12,15]–a value partly dependent on the type of assay used [22]. Because there appears to be a close relationship between disease activity and ATI/ITL in CD [16, 23], it has been proposed that a management strategy based on ATI/ITL measurements might be more efficient and cost effective than one based on clinical parameters [24, 25].

To address these issues we have studied here the impact of ATI and ITL in a subgroup of those patients in clinical (CDAI <150) and endoscopic (Rutgeerts score 0–1) remission after 12 months of therapy with infliximab 3 mg/Kg. In addition to ITL and ATI, putative markers of disease activity/inflammation—HS-CRP and FC—were also measured during the 8-week therapeutic interval. For comparison purposes ITL, ATI and inflammatory markers in the study subjects were contrasted to those of control patients—i.e. CD patients maintained in clinical remission (CDAI <150) with infliximab 5 mg/Kg.

In both controls and study subjects the levels of the inflammatory markers appeared in a dynamic balance with infliximab concentrations throughout the 8-week interval. FC and HS-CRP appeared on the average higher in controls than in study subjects at any time point, suggesting a tighter control of inflammation in the latter.

ITL were detectable in all patients treated with infliximab 3 mg/Kg but were below the hypothetical therapeutic threshold of 3 μg/ml in 4 out of 5 patients. By contrast and as expected, ITL were greater than 3 μg/ml in all the controls. Low levels ATI were detected in 2 out of 5 patients treated with low infliximab doses, while being absent in all the controls. Patients on low dose infliximab continued such treatment and underwent colonoscopy after 18 additional months of therapy on the average. Colonoscopy was unchanged compared to baseline—i.e. all patients remained in full endoscopic remission. Likewise, CDAI remained below 150 in all study subjects after a total of 30 months of treatment. No infusion reactions or infliximab-related side effects were recorded within the entire follow-up period.

Overall, these data show that an infliximab dose of 3 mg/kg appears sufficient to avoid endoscopic and clinical POR in the long term. Our data also suggest that the threshold therapeutic ITL might be lower than 3 μg/ml in relapsing patients after surgery. In patients treated with infliximab 3 mg/Kg, ATI levels—when present—were <10 U/mL, a concentration which may not affect clinical outcomes [21,22] possibly because of their transient nature [21].

Our study has many obvious limitations, including the very small sample size and the measurement of ATI during a limited period of time. In addition, this is the follow up of an original study which was not blinded or randomized. Nevertheless, each patient was thoroughly studied clinically, endoscopically and biochemically. Our study compared drug pharmacokinetics and inflammatory markers of post-operative CD patients treated with low infliximab dose with those of CD patients who did not undergo surgery in remission on 5 mg/Kg infliximab. It was not an aim of this study to compare the clinical and endoscopic outcomes between the two different groups—which conceivably differed in terms of disease burden and therapeutic needs. Rather, we included patients treated with the standard infliximab dose as an internal control—since CD patients on 5 mg/Kg in full clinical remission have usually ITL > 3 μg/ml and are ATI negative [12,15], findings confirmed by our study.

It is possible that low infliximab doses might also be able to control inflammation in a proportion of CD patients who have not undergone surgery but bear a low disease burden like those operated on. Clearly, the comparative effectiveness of such strategy in both groups would need to be confirmed in a large study.

In conclusion, our small study provides objective support to the hypothesis that infliximab doses as low as 3 mg/Kg might be effective in the long term to prevent disease recurrence in patients undergone surgery for CD. For the time being, it is difficult to envision more efficient long term strategies when using anti-TNF agents. As in CD patients who have not had surgery, it is possible that a proportion of patients with POR on combination therapy might be kept in clinical remission by stopping infliximab while maintaining the immunomodulator [10]. In such case, it would remain to be seen whether the addition of an immunomodulator to anti-TNF agents in the postoperative setting would bear clear advantages to justify the known risk of side effects in patients exposed to combination therapy [26].
